# Supplementary material for: The interactome of intact mitochondria by cross-linking mass spectrometry provides evidence for coexisting respiratory supercomplexes
Source: Mol Cell Proteomics. 2017 Dec 8;17(2):216–32. doi: 10.1074/mcp.RA117.000470 (PMC5795388; doi:10.1074/mcp.RA117.000470)
Supplement: Supplemental Data [file supp_RA117.000470_133922_0_supp_23978_fzffwf.zip › spectra_annotation/mito_spectra_annotation/73-1-1-1-9-1.pdf]

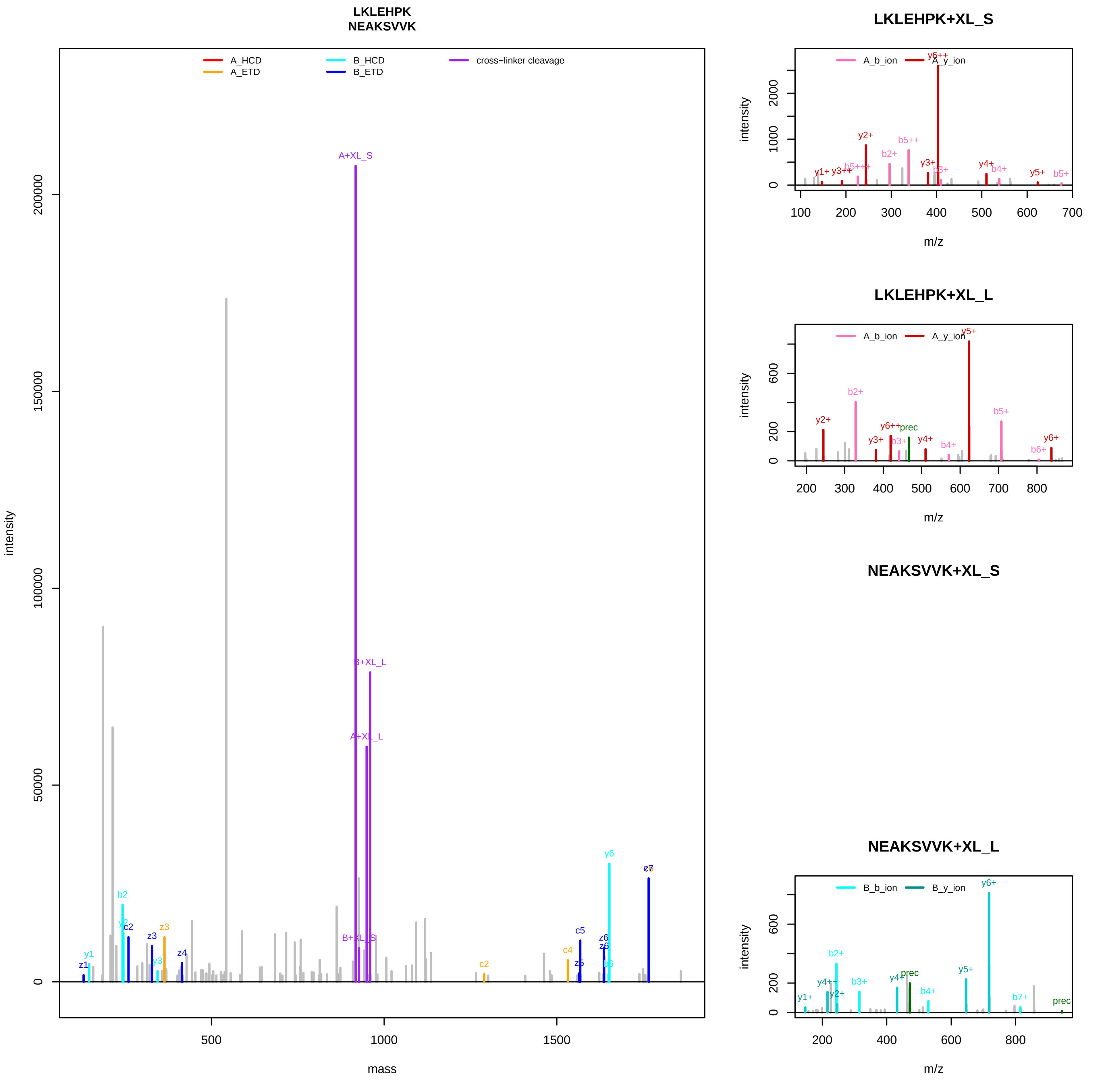

LKLEHPK
NEAKSVVK
A_HCD
A_ETD
B_HCD
B_ETD
cross−linker cleavage
intensity
mass
0
50000
100000
150000
200000
500
1000
1500
A+XL_S
B+XL_L
A+XL_L
B+XL_S
z1
y1
b2
y2
c2
z3
y3
z3
z4
c2
c4
c5
z5
z6
z6
y6
b6
c7
LKLEHPK+XL_S
A_b_ion
A_y_ion
y6++
y2+
b5++
b2+
y3+
b3+
y4+
b4+
y5+
b5+
y1+
y3++
b5+++
m/z
LKLEHPK+XL_L
y5+
b2+
y2+
y6++
prec
y3+
b3+
y4+
b4+
b5+
b6+
y6+
NEAKSVVK+XL_S
NEAKSVVK+XL_L
B_b_ion
B_y_ion
y6+
b2+
y4++
y2+
b3+
y4+
prec
b4+
y5+
b7+
y1+
